# Comparative Evaluation of Lipid Profile, C-Reactive Protein and Paraoxonase-1 Activity in Dogs with Inflammatory Protein-Losing Enteropathy and Healthy Dogs

**DOI:** 10.3390/ani14213119

**Published:** 2024-10-29

**Authors:** Paola Gianella, Federica Cagnasso, Alessia Giordano, Antonio Borrelli, Enrico Bottero, Barbara Bruno, Riccardo Ferriani, Franca Borella, Sara Meazzi, Donatella Scavone, Saverio Paltrinieri

**Affiliations:** 1 Department of Veterinary Sciences, University of Turin, Largo P. Braccini 2, 10095 Grugliasco, Italy; federica.cagnasso@unito.it (F.C.); antonio.borrelli@unito.it (A.B.); franca.borella@unito.it (F.B.); 2 Department of Veterinary Medicine and Animal Sciences, University of Milan, Via dell’Università 6, 26900 Lodi, Italy; alessia.giordano@unimi.it (A.G.); sara.meazzi@i-vet.it (S.M.); donatella.scavone@guest.unimi.it (D.S.); saverio.paltrinieri@unimi.it (S.P.); 3 Endovet Italia, Via A. Oroboni, 00100 Roma, Italy; botvet@libero.it (E.B.); riccardo.ferriani@libero.it (R.F.)

**Keywords:** canine, chronic enteropathy, cholesterol, CRP, IBD, lipoprotein, PLE, PON-1, triglycerides

## Abstract

Chronic inflammation alters lipoprotein metabolism and causes changes in the serum concentrations of lipids, C-reactive protein (CRP), and paraoxonase-1 activity (PON-1), an enzyme that may act as a local detoxifier, antioxidant, and immunomodulator in the gastrointestinal tract. Scarce information is available in dogs with protein-losing enteropathy secondary to chronic enteropathy (iPLE). The first aim was to describe and compare the lipid profiles, CRP concentrations and PON-1 activities in healthy dogs and in dogs with iPLE. The second aim was to evaluate correlations among clinicopathological, histologic data and lipid profiles in dogs with iPLE. Serum samples from 51 iPLE and 40 healthy dogs were used to study albumin, total protein, CRP, PON-1 activity, cholesterol, triglycerides and lipoprotein classes. Serum concentrations of albumin, total protein, cholesterol, PON-1 activity, and high-density and very-low-density lipoproteins were lower in iPLE dogs compared to healthy controls, while those of triglycerides, low-density lipoproteins, chylomicrons and CRP were higher. Significant correlations between the lipid profile and the existing chronic enteropathy activity index were not found. High-density and low-density lipoproteins correlated with CRP and PON-1. Triglycerides were significantly higher in dogs with both inflammation and lymphangiectasia. The results need to be confirmed in further studies.

## Simple Summary

Various changes in lipid profile have been described in human patients with inflammatory bowel disease, in addition to alterations in C-reactive protein (CRP) concentration, and paraoxonase-1 (PON-1) activity, an antioxidant enzyme that may be responsible for some alterations in the lipoprotein profile. Since lipoproteins, C-reactive protein and paraoxonase-1 take part in inflammation, investigating their changes in dogs with protein-losing enteropathy secondary to chronic inflammatory enteropathy (iPLE) is crucial. In addition, correlations among clinicopathologic data, histopathological findings and the lipid profile were evaluated. Left-over serum samples from healthy dogs and dogs with iPLE were used, and concentrations of C-reactive protein, triglycerides, cholesterol, lipoproteins and paraoxonase-1 activity were studied. Dogs with iPLE had altered concentrations of all analytes. Moreover, some correlations among clinicopathologic data, histopathological findings and the lipid profile were found. These findings suggest that dyslipidemia may be observed in dogs with iPLE. Further studies are needed to provide more information on this subject.

## 1. Introduction

Canine protein-losing enteropathy (PLE) is a syndrome characterized by an abnormal loss of serum proteins through the gastrointestinal mucosa [1,2,3]. Numerous gastrointestinal diseases such as intestinal lymphangiectasia, lymphoma, regional fungal infections, and chronic inflammatory enteropathy, if severe enough, can lead to PLE [1,2,3]. In people, PLE is usually associated with primary intestinal lymphangiectasia (IL) [4]. In dogs, although a genetic susceptibility to the development of primary IL has been reported for some breeds, PLE is more commonly associated with secondary IL resulting from chronic enteropathy (CE) [1,2,3,5,6,7,8,9]. Since lymphatics are the primary transporters of lipids, lipid-soluble vitamins, food antigens, bacteria-derived lipopolysaccharides, and gut hormones from the intestine to the blood, their dysfunction can contribute to the pathogenesis and progression of intestinal inflammation [10]. The diagnostic workup of dogs with PLE is similar to that of dogs with CE [2]. Although dietary therapy (often with low-fat or ultra-low-fat formulations) alone is associated with a positive outcome, glucocorticoids (at anti-inflammatory or immunosuppressive dosages), immunosuppressive agents and supportive therapies are needed in some dogs with PLE [2,7,9,11,12,13,14,15,16]. Although prolonged survival can occur, PLE is often characterized by a guarded prognosis and high rate of relapse [1,2,17,18,19].

Various lipid-profile changes have been described in human patients with inflammatory bowel disease and could be mainly summarized by decreased levels of total cholesterol (Chol) and low-density lipoproteins (LDLs), variable levels of high-density lipoproteins (HDLs), and normal or increased levels of triglycerides (TGs) [20,21,22,23]. These changes are thought to be the result of a complex interaction of inflammatory cytokines with the down-regulation of the lipolytic enzyme activity, malnutrition and lipid malabsorption [24,25]. In addition, total and HDL-cholesterol levels are correlated with the systemic inflammatory status [26]; indeed, interleukin-6, a pro-inflammatory cytokine, and C-reactive protein (CRP), a biomarker of systemic inflammation, are closely related and together play a role in general lipid metabolism, inhibiting adipocyte lipoprotein lipase activity [27]. Finally, additional mediators such as paraoxonase-1 (PON-1), an HDL-bound antioxidant enzyme, which may act as a local detoxifier, antioxidant, and immunomodulator in the gastrointestinal tract, may be responsible for some changes within the lipid profile [28,29].

In veterinary medicine, serum and plasma lipoprotein profiles have been sporadically investigated both in healthy and diseased dogs [30,31,32,33,34,35]. Among healthy dogs, HDLs are commonly the predominant lipoprotein fraction, while lower percentages of LDLs and very-low-density lipoproteins (VLDLs) are observed [36]. Among diseased dogs, the percentage of HDLs decreases in chronic kidney disease, nephrotic syndrome, babesiosis, leishmaniasis and pancreatitis; meanwhile, the percentage of VLDLs, LDLs and chylomicrons increases in brachycephalic syndrome, chronic kidney disease, nephrotic syndrome, diabetes mellitus, sepsis and pancreatitis [31,32,33,34,37]. However, to the best of the authors’ knowledge, no information exists on the lipid profile in dogs with PLE secondary to chronic enteropathy (iPLE). CRP, a nonspecific marker of inflammation, has been used in CE and iPLE to select the best clinical approach at the onset of treatment, and to document disease progression and the response to treatment [38,39,40,41]. Low serum PON-1 activity concentrations have already been demonstrated in some dogs with both acute and chronic inflammation, likely because an intense oxidation occurs [42,43]. However, to the best of the authors’ knowledge, no information exists on PON-1 activity in dogs with iPLE. 

Based on these premises, the study aimed (i) to describe and compare the lipid profile, CRP and PON-1 activity between healthy dogs and dogs with iPLE, and (ii) to evaluate associations among clinicopathological data, histopathological findings and the lipid profile in dogs with iPLE. We hypothesized that in dogs with iPLE, the lipid profile, CRP and PON-1 activity would be altered and that lipid profile would be varied as a function of disease severity.

## 2. Materials and Methods

### 2.1. Animals

This prospective study was conducted on 51 serum samples from privately owned dogs that were newly diagnosed with iPLE from January 2021 to March 2022 at the Veterinary Teaching Hospital, Department of Veterinary Sciences, University of Turin, Italy, and other referral clinics in northern and central Italy. In addition, a total of 40 serum samples from healthy dogs were used. The study received the official approval of the Institutional Ethics and Animal Welfare Committee (protocol number 42/2021).

The criteria for the diagnosis of iPLE were as follows: chronic gastrointestinal signs lasting for more than 3 weeks, hypoalbuminemia of gastrointestinal origin (≤2.8 g/dL) and histopathological evidence of benign gastrointestinal inflammation with or without lymphangiectasia on multiple biopsies collected by endoscopy [1,2,3,5,8]. A histopathologic examination, conducted in accordance with the histopathological standards of the World Small Animal Veterinary Association Gastrointestinal Standardization Group, was required [44]. Extra-intestinal diseases, liver and pancreatic diseases, infectious or parasitic diseases and intestinal diseases of other etiology (e.g., intussusception, foreign body or intestinal tumor) were excluded through the results of the following diagnostic investigations: clinical evaluation, full abdominal ultrasound, hematobiochemical panel (including complete blood count, electrolytes, albumin, total protein, globulin, symmetric dimethylarginine, creatinine, urea, glucose, total bilirubin, cholesterol, triglycerides, alanine aminotransferase, alkaline phosphatase, aspartate aminotransferase, gamma-glutamyl transferase, creatine kinase, fructosamine), fecal flotation and Giardia antigen test, pre- and post-prandial bile acids, urinalysis, urinary protein-to-creatinine ratio, serum basal cortisol or ACTH stimulation test (if basal cortisol ≤ 2 µg/dL), trypsin-like immunoreactivity, pancreas-specific lipase levels, serum folate and cobalamin concentrations. In particular, a negative fecal flotation and Giardia antigen test, normal pre- and post-prandial bile acid concentration, basal cortisol >2 µg/dL or appropriate cortisol levels after ACTH stimulation, and serum concentrations of TLI and pancreas-specific lipase within the reference interval were required. In order to confirm the gastrointestinal origin of hypoalbuminemia, dogs were also required to have no clinically relevant proteinuria (negative urine dipstick test result or urine protein-to-creatinine ratio ≤0.5) and no evidence of clinically relevant hepatic disease (normal pre- and post-prandial bile acid concentrations or normal synthetic liver function and enzyme activity). All dogs had to be fed nutritionally complete and balanced commercial or home-cooked diets. A minimum of 12 h of fasting were required before blood collection. Exclusion criteria were complete and sustained response to dietary trials and gut microbiota manipulation (i.e., pre-, pro-, syn- and postbiotics, fecal microbiota transplantation and antibiotics), histopathologic diagnosis of neoplasia and treatment with antibiotics or immunosuppressive drugs within 1 month of study enrollment. All dogs underwent gastroduodenoscopy. Colonoscopy with ileal intubation was performed when possible. Biopsies from the stomach, duodenum and, when available, the ileum and colon, were collected for histologic examination. The severity of morphologic and inflammatory lesions in the duodenum, ileum and colon were recorded as follows: 0 = normal, 1 = mild, 2 = moderate, 3 = marked [44]. The mean cumulative lesion score calculated as the sum of individual lesion scores from each segment was considered for statistical analysis. Each dog was further assigned to group 1 or group 2 based on the presence of inflammation with only mild or no lacteal dilation, and inflammation with moderate or severe lacteal dilation, respectively.

Information gathered from the medical records on signalment, body weight (kg), the body condition score (BCS, scale 1–9) [45] and the canine chronic enteropathy clinical activity index (CCECAI) score [46] was studied, in addition to the type of diet. The CCECAI score was calculated using the serum albumin concentration, presence or absence of peripheral edema and peritoneal effusion on ultrasound examination, and the owner’s scores on appetite, activity level, vomiting, fecal consistency and frequency, weight loss and pruritus. Four disease severity groups were identified based on the CCECAI scores: mild disease (CCECAI 4-5; MI), moderate disease (CCECAI 6–8; MO), severe disease (CCECAI 9–11; S) and very severe disease (CCECAI ≥ 12; VS) [46].

The control group included healthy owned dogs, aged 1 year old and above, that were regularly vaccinated and receiving appropriate ecto-and endo-parasite preventive treatment. All healthy dogs were belonging to staff at the Veterinary Teaching Hospital, Department of Veterinary Sciences, University of Turin, Italy, or were presented at the same facilities of the study group for their annual check-up and vaccination. All healthy dogs had to be fed nutritionally complete and balanced diets (commercial or home-cooked). Dogs were considered healthy based on an unremarkable history and physical examination (allowed BCS 4-6), hematobiochemical panel (including complete blood count, electrolytes, albumin, total protein, globulin, symmetric dimethylarginine, creatinine, urea, glucose, total bilirubin, cholesterol, triglycerides, alanine aminotransferase, alkaline phosphatase, aspartate aminotransferase, gamma-glutamyl transferase, creatine kinase, fructosamine), negative fecal flotation, and the absence of any gastrointestinal sign within one year prior to enrollment. In addition, there was no history of drug administration in the 6 months prior. Finally, a minimum of 12 h of fasting was required before blood collection. 

### 2.2. Sample Collection, Lipid Profile, CRP and PON-1 Activity

The serum left-over samples obtained from centrifugation of blood collected from each dog for diagnostic purposes on admission and after 12 h of fasting were separated and immediately stored at −80 °C until analysis. Sample storage varied from 3 to 18 months. 

All analyses were performed at the clinical pathology laboratory of the Veterinary Teaching Hospital of the University of Milan, Lodi, Italy. The evaluation of the lipid profile included Chol, TGs and lipoprotein classes. Frozen serum samples were thawed and used to measure serum concentrations of albumin (Alb), total proteins (TP), Chol, TGs and CRP. All of these analytes were measured on the automated chemistry analyzer BT 3500 (Biotecnica Instruments SPA, Rome, Italy) using reagents and methods provided by Futurlab Srl (Limena, PD, Italy). Specifically, Chol and TGs were measured with the colorimetric enzymatic CHOD-PAP and GPO-PAP methods, respectively. PON-1 activity was also measured on the same instrument, using the reagent and method validated in dogs, as previously described [42]. Based on the serum albumin concentration, dogs were further assigned to group A (2.2 to 2.8 g/dL), B (1.5 to 2.19 g/dL), C (1.2 to 1.49 g/dL) or D (<1.2 g/dL). Lipoprotein analysis was carried out on the same serum samples on buffered (pH 8.5) agarose gel with a semi-automated instrument (Hydrasis, Sebia Italia S.r.l., Bagno a Ripoli, Italy), using kits produced by the manufacturer (Hydragel 15 lipoproteins). After migration (160 V, 25 min), agarose gels (8 g/L) were stained with Sudan black, washed with ethanol (45%), dried and placed on the gel scanner for the densitometric analysis. Scanned images were analyzed using the software Phoresis (Sebia Italia S.r.l., Bagno a Ripoli, Italy) that calculates the area under the peaks corresponding to HDLs, VLDLs, LDLs and chylomicrons, and expresses the results as a percentage of the total area (HDL%, VLDL%, LDL%, chylomicrons%) [47].

### 2.3. Statistical Analysis

All data were analyzed with the software GraphPad Prism 9.5.1 (Dotmatics). Significance was set at *p* < 0.05. Data were tested for normality by the Shapiro–Wilk test. Data were reported as the median, minimum and maximum. Data were compared between healthy and diseased dogs by the use of the Student’s *t*-test in the case of normal distribution and the Mann–Whitney U test in the case of a non-normal distribution. All comparisons among groups were performed by use of the one-way ANOVA test or Kruskal–Wallis test dependent on the normality test. The *p*-values were adjusted with the Dunn’s multiple comparison test. Due to the low numerosity, data from groups C and D were merged into a single group (C + D). Correlations among iPLE dogs were assessed by use of the Spearman correlation test.

## 3. Results

### 3.1. Patient Data 

Among the healthy control dogs, 24 (13 spayed) were female and 16 (7 neutered) were male. Seven dogs were of mixed breed (17.5%) and thirty-three were purebred (82.5%), represented as follows: Australian Shepherd, English Setter and Yorkshire Terrier (four dogs of each breed); Labrador Retriever and German Shepherd (three dogs of each breed); Alpenlaendische Dachsbracke and Dachshund (two dogs of each breed); and Podenco ibicenco, Jack Russel Terrier, Springer Spaniel, Chihuahua, Pitbull, Spanish Greyhound, Cavalier King Charles, Golden Retriever, Pug, Border Collie and Rottweiler (one dog of each breed). The median age was 48 months (range 12–208), the median body weight was 15.2 kg (range 2.5–42), the median BCS was 5 (range 4–6) and the muscle condition score was graded as normal in all dogs. Thirty-six healthy control dogs were fed commercial, nutritionally complete and balanced canine diets of different brands. The remaining four healthy dogs were fed home-cooked nutritionally complete and balanced canine diets.

Among the dogs with iPLE, 20 (39.2%) were female (15 spayed) and 31 (60.8%) were male (2 neutered). Eight dogs were mixed-breed (15.7%) and forty-three dogs were purebred (84.3%), represented as follows: German Shepherd (nine dogs); Golden Retriever, English Setter and Yorkshire Terrier (three dogs of each breed); Australian Shepherd, Border Collie, Chihuahua, Labrador Retriever, Maltese Dog and Spanish Greyhound (two dogs each breed); and American Staffordshire Terrier, Belgian Shepherd, Boston Terrier, Cavalier King Charles, Cesky Terrier, Cocker Spaniel, Dachshund, Doberman Pinscher, Jack Russell Terrier, Pitbull, Podenco ibicenco, Pug and Rottweiler (one dog each breed). The median age was 84 months (range 19–171), median body weight was 15 kg (range 2.4–47.5), median CCECAI score was 9 (range 3–17) and median BCS was 3 (range 1–6). Six, 19, 13 and 13 dogs were assigned to group MI, MO, S and VS severity subgroup, respectively. Based on the concentrations of serum albumin, 6 dogs (11.8%) were assigned to group A, 29 (56.9%) to group B, 12 (23.5%) to group C and 4 (7.8%) to group D. Sex and body weight did not significantly differ between healthy and diseased dogs. BCS was significantly lower in the iPLE dogs compared to the healthy dogs (*p* < 0.0001). Age was significantly higher in the iPLE dogs compared to the healthy dogs (*p* < 0.0001). All dogs had gastrointestinal duodenoscopy performed. Thirty-four dogs (66.7%) had concurrent lower GI endoscopy, in which the ileum was successfully intubated in 11 dogs (21.6%). On histopathology, a predominantly lymphoplasmacytic infiltration of the intestinal mucosa was found in all dogs. With regard to the histologic lesion severity, mild (grade 1) duodenal, ileal and colonic histologic lesions were found in 0, 0 and 4 dogs, respectively; moderate (grade 2) duodenal, ileal and colonic histologic lesions were found in 25, 9 and 29 dogs, respectively; marked (grade 3) duodenal, ileal and colonic histologic lesions were found in 26, 2 and 1 dog, respectively. Dilated crypts with proteinaceous material and cellular debris (crypt abscesses) were identified in 10 dogs (19.6%), and they were further classified as mild (*n* = 4; 7.8%), moderate (*n* = 3; 5.9%) or marked (*n* = 3; 5.9%). Lacteal dilation was identified in 40 dogs (78.4%), and it was further classified as mild (*n* = 17, 42.5%), moderate (*n* = 22, 55%) or marked (*n* = 1, 2.5%). Twenty-seven dogs were further assigned to group 1 (inflammation with only mild or no lacteal dilation) and twenty-four to group 2 (inflammation with moderate or severe lacteal dilation). On admission, 12 dogs were on highly digestible gastrointestinal commercial diets, 3 were on highly digestible low-fat commercial diets, 15 were on limited-ingredient commercial diets, 9 were on home-cooked low-fat diets and 8 were on hydrolyzed diets. Four dogs were fed different diet types. No dogs were receiving prednisolone at the time of blood sampling. However, in the month before referral, 11 dogs received 0.5 mg/kg SID of prednisolone for 4 days, 1 dog received 0.5 mg/kg SID for 3 days twice in a 3-week period and 1 dog received 1 mg/kg SID for 3 days.

### 3.2. Comparative Evaluation of the Lipid Profiles and Other Laboratory Parameters Between Healthy Control Dogs and iPLE Dogs

**Figure 1:**
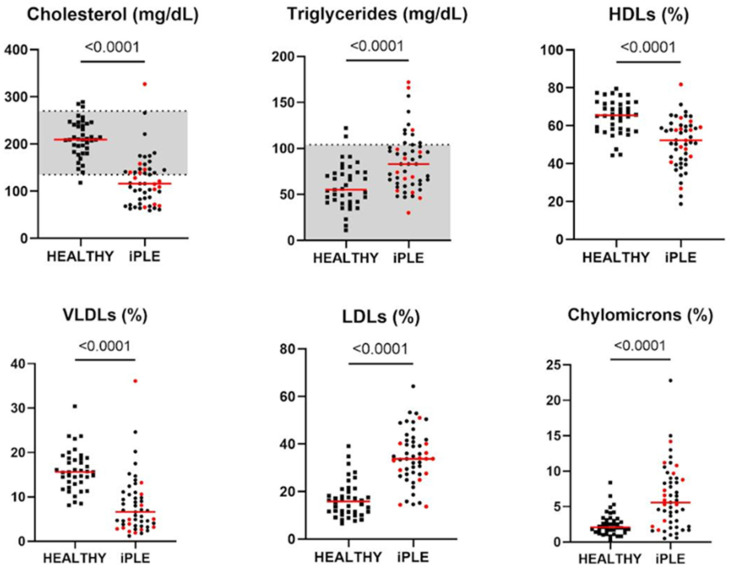
Serum concentrations of cholesterol, triglycerides, HDL, VLDL, LDL and chylomicron lipoprotein classes in healthy control dogs and iPLE dogs. Red lines indicate the median value. The horizontal bars delimit the minimum and maximum reference values. Red dots indicate dogs that received prednisolone before admission. iPLE = inflammatory protein-losing enteropathy; HDLs = high-density lipoproteins; LDLs = low-density lipoproteins; VLDLs = very-low-density lipoproteins.

The results recorded in healthy control and iPLE dogs are reported in Table 1 and, with regard to the lipid profile, in Figure 1. In addition, an example of the lipoprotein gel analysis of both healthy and iPLE dogs is reported as Supplemental data (Figure S1). Among the iPLE dogs, 30 and 39 showed decreased Chol and PON-1 activity, respectively, while 26 and 13 showed increased CRP and TGs, respectively. Serum concentrations of Alb, TP, Chol, HDLs, VLDLs and PON-1 activity were significantly lower in iPLE dogs compared to healthy control dogs. Serum concentrations of TGs, LDLs, chylomicrons and CRP were significantly higher in iPLE dogs compared to healthy control dogs.

### 3.3. Correlations of the CRP Concentrations with CCECAI Scores and Histopathological Findings

A positive weak correlation between CCECAI scores and CRP (*r* = 0.28, *p* = 0.042) was found. The mean cumulative lesion score did not correlate with CRP. No significant difference in CRP was found between group 1 (inflammation with only mild or no lacteal dilation) and 2 (inflammation with moderate or severe lacteal dilation).

### 3.4. Correlations of the Lipid Profile with Age, Gender, Body Weight, BCS, CCECAI Score, Alb, CRP and PON-1 Activity Concentrations

Significant correlations of the lipid profile and age, body weight, PON-1 activity and CRP are summarized in Table 2. Significant correlations between the lipid profile and the BCS were not found. Significant correlations between the lipid profile and the CCECAI score were not found. Significant correlations between the lipid profile and Alb were not found. The results of all correlations studied are reported in a Supplemental Table (Table S1).

### 3.5. Comparisons of the Lipid Profile with CCECAI Disease Severity Groups and Hypoalbuminemia Groups

**Figure 2:**
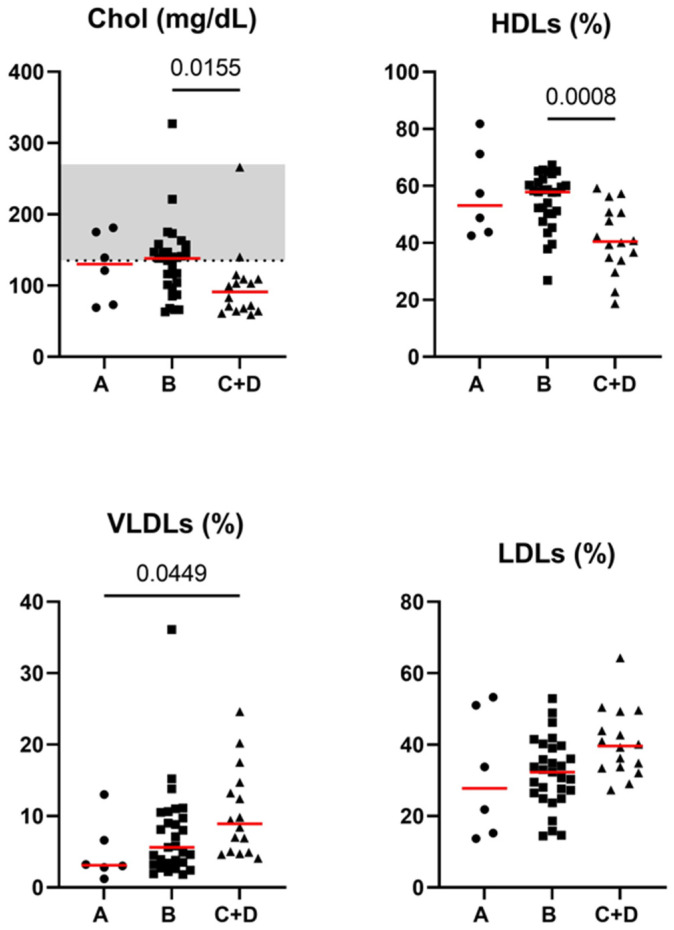
Serum concentrations of cholesterol, HDL, LDL and VLDL lipoprotein classes in iPLE dogs assigned to hypoalbuminemia groups (A, B, C + D). Group A (serum albumin concentration 2.2 to 2.8 d/dL), group B (serum albumin concentration 1.5 to 2.19 g/dL), group C (serum albumin concentration 1.2 to 1.49 g/dL) and group D (serum albumin concentration < 1.2 d/dL). Red lines indicate the median value. The horizontal bars delimit the minimum and maximum reference values. Chol = cholesterol; iPLE = inflammatory protein-losing enteropathy; HDLs = high-density lipoproteins; LDLs = low-density lipoproteins; VLDLs = very-low-density lipoproteins. Dogs belonging to group A have been indicated with a circle. Dogs belonging to group B have been indicated with a square. Dogs belonging to group C + D have been indicated with a triangle.

Significant differences in the lipid profiles among the CCECAI disease severity groups were not found. When considering the hypoalbuminemia groups, some significant differences regarding Chol (*p* = 0.015), HDLs (*p* = 0.001), VLDLs (*p* = 0.026) and LDLs (*p* = 0.048) were found. More specifically, Chol and HDLs were significantly lower in group C + D compared to group B (adjusted *p* = 0.015 and adjusted *p* = 0.0008, respectively), and VLDLs were significantly higher in group C + D compared to group A (adjusted *p* = 0.045). LDLs did not significantly differ in terms of Dunn’s multiple comparison test. Serum concentrations of Chol, HDL, LDL and VLDL lipoprotein classes in iPLE dogs classified by hypoalbuminemia groups are reported in Figure 2. The results of all the comparisons studied are reported in a Supplemental Table (Table S1).

### 3.6. Comparisons of the CRP and PON-1 Activity Concentrations with Hypoalbuminemia Groups

**Figure 3:**
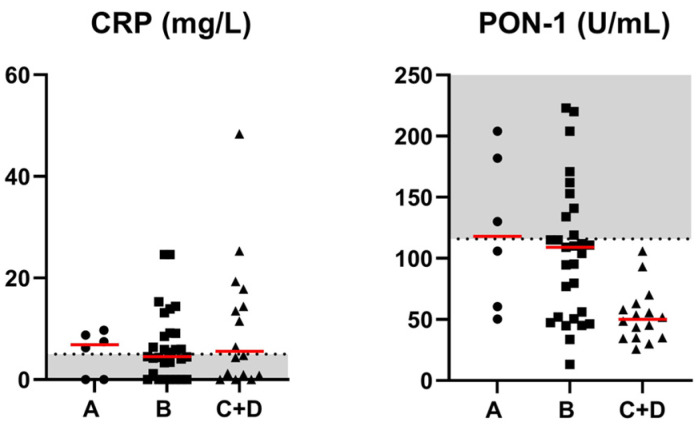
Serum concentrations of CRP and PON-1 activity in iPLE dogs classified by hypoalbuminemia groups (A, B, C + D). Group A (serum albumin concentration 2.2 to 2.8 d/dL), group B (serum albumin concentration 1.5 to 2.19 g/dL), group C (serum albumin concentration 1.2 to 1.49 g/dL) and group D (serum albumin concentration < 1.2 d/dL). Red lines indicate the median value. The horizontal bars delimit the minimum and maximum reference values. CRP = C-reactive protein; iPLE = inflammatory protein-losing enteropathy; PON-1 = paraoxonase-1. Dogs belonging to group A have been indicated with a circle. Dogs belonging to group B have been indicated with a square. Dogs belonging to group C + D have been indicated with a triangle.

The serum concentration of CRP was increased in 4, 14, 5 and 3 dogs of group A, B, C and D, respectively. The serum concentration of PON-1 activity was decreased in 3, 20, 12 and 4 dogs of group A, B, C and D, respectively. Significant differences among groups between CRP concentration and hypoalbuminemia groups were not found. Significant differences among groups between PON-1 concentration and hypoalbuminemia groups were found (*p* = 0.0018). More specifically, the PON-1 concentration was significantly lower in group C + D compared to group A (adjusted *p* = 0.020), and in group C + D compared to group B (adjusted *p* = 0.004). Serum concentrations of CRP and PON-1 activity in iPLE dogs classified by hypoalbuminemia groups are reported in Figure 3.

### 3.7. Correlations of the Lipid Profile with Histopathological Findings and Diet

No significant correlations between the lipid profile and the mean cumulative lesion score were found. By comparison of the lipid profile between dogs of group 1 (inflammation with only or no lacteal dilation) and group 2 (inflammation with moderate or severe lacteal dilation), TGs were significantly higher in the dogs of group 2 (*p* = 0.0045). 

No significant differences in the lipid profile among dogs classified based on the type of diet upon admission (hydrolyzed diets, highly digestible gastrointestinal commercial diets, limited-ingredient commercial diets, home-cooked low-fat diets) were found.

### 3.8. Comparative Evaluation of the Lipid Profile and Other Selected Variables Between iPLE Dogs That Received Prednisolone and iPLE Dogs That Did Not

Results recorded in iPLE dogs that received prednisolone before admission and iPLE dogs that did not are reported in Table 3. Based on the CCECAI severity groups, two dogs that received prednisolone before admission were assigned to group MI, four to group MO, four to group S, and three to group VS. Among dogs that did not, 4 were assigned to group MI, 15 to group MO, 9 to group S, and 10 to group VS. Based on the concentrations of serum albumin, three dogs that received prednisolone before admission were assigned to group A, seven to group B, two to group C, and one to group D. Among dogs that did not, 3 were assigned to group A, 22 to group B, 10 to group C and 3 to group D. No significant differences in age, body weight, BCS, CCECAI score, Alb, CRP and PON-1 activity concentrations, and lipid profile were found between the two groups. 

## 4. Discussion

We described and compared for the first time, the lipid profile, CRP and PON-1 activity between healthy control dogs and dogs with iPLE, and explored associations among clinicopathological data, histopathological findings and the lipid profile in dogs with iPLE.

Hypoalbuminemia is the hallmark of the PLE syndrome in dogs; however, hypocholesterolemia can be observed and it can help in define the prognosis [1,7,48]. To the authors’ knowledge and unlike in people, scarce or no information is available on serum TGs and lipoprotein changes in dogs with CE [49]. Recently, the lipoprotein profile of cavitary effusions was investigated in some dogs with PLE, but the same analysis was not performed on serum samples [49]. Transudates of dogs with PLE were found to be poor in protein, cholesterol and HDL contents, and high in VLDL and LDL contents [49]. To date, the diagnosis of canine dyslipidemia is based on the measurement of pre-prandial serum cholesterol and triglyceride concentrations, while the lipoprotein analysis is not routinely used. Some explanations can be attempted. A gold-standard validated method for the evaluation of canine lipoproteins is currently lacking; different techniques can be used, some of them developed for human use, but they are expensive, time and labor consuming or not suitable for canine lipoprotein analysis [30,32,36,37,49,50,51,52]. Accordingly, information concerning reliable reference intervals for the different lipoprotein classes is lacking, and the results are poorly reproducible and not always comparable. Agarose gel electrophoresis is used to classify lipoproteins by the nomenclature beta, pre-beta and alpha, based on the mobility of LDLs, VLDLs and HDLs, respectively [53]. This method was selected here because it provides accurate separation of canine lipoproteins, and it might be superior to the wet chemistry method for identifying some lipoprotein classes, especially LDLs, which are substantial in some dogs [30,35,50,54]. However, it has not been extensively investigated for use in canine gastroenterology. 

In both healthy control dogs and iPLE dogs, the HDL lipoprotein class was predominant, followed by the LDL and VLDL lipoprotein classes. According to the available information, these results are not surprising since HDLs represents the predominant lipoprotein class in dogs, whereas in humans, the LDL class predominates [55]. On the other hand, there are neither reports regarding lipoprotein profiles in dogs with iPLE, nor published solid reference intervals obtained by agarose gel electrophoresis to be used here for comparison. Hypocholesterolemia, variable levels of HDLs and LDLs, and normal or increased levels of TGs are common findings in humans with active IBD [20,21,22,23,26,56,57]. Moreover, some of these findings are independently associated with more severe disease [23]. Similarly, our iPLE dogs showed significantly decreased serum concentrations of Chol, and percentages of HDLs and VLDLs compared to healthy controls, while concentrations of chylomicrons and TGs were normal or increased [26]. These results might suggest that lipid metabolism is also affected in dogs with iPLE. However, it cannot be ruled out that some dogs with normal TGs have had hypotriglyceridemia, since there is no lower reference limit for TGs. Hypocholesterolemia, presumably secondary to inflammation, lymphangiectasia, fat malabsorption and malnutrition, has been already documented among dogs with iPLE, while scattered information is available for serum TGs [7,11,58]. Hypocholesterolemia is a common feature in human patients with acute diseases, and it has been related to surrogate markers of disease severity including IBD-related surgeries and the number of hospitalizations [59,60]. Hyperchylomicronemia is unexpected, since iPLE dogs have difficulty absorbing fat, and it is dietary fat that is used to form chylomicrons. Two hypotheses can be attempted. First, chylomicrons may not be quantitatively different between the two groups, despite the presence of a significant difference in their percentage values. Indeed, the results of the lipoprotein gel electrophoresis are expressed as a percentage of the total area, and the decrease in a lipoprotein class determines the increase in another class. Second, recent evidence suggests that postprandial chylomicron output and transport through intestinal lymphatics are not impaired in IBD patients [61]. It cannot be excluded that similar mechanisms, along with lymphangiogenesis and other stimuli of intestinal lipid mobilization, take place also in some dogs with iPLE, contributing to the increase in chylomicrons [62]. However, this remains speculative at this time and further studies are needed.

In this study, no significant correlations were found between disease severity, as assessed by the CCECAI score, and the lipid profile. Some hypotheses may be attempted. The CCECAI score, although routinely used to assess the clinical severity of canine CE, might not be appropriate to identify subsets of dogs with severe intestinal inflammation and lipid malabsorption. Moreover, the severity of lipid malabsorption could not play a pivotal role in the clinical manifestations in some dogs. 

Similar to previous results, CRP was significantly increased in iPLE dogs when compared with healthy dogs [63,64]. Our results were also in accordance with previously published results showing a correlation between CRP and clinical severity scores in dogs with CE [63]. However, no correlations or differences among groups between CRP concentration and histopathological findings or ongoing prednisolone therapy on admission in iPLE dogs were found. Therefore, in some dogs with iPLE, CRP might be not useful for assessing the histopathologic severity of inflammation and lacteal dilation, nor for assessing the response to treatment, in contrast with previous observations [63]. This discrepancy, however, might be because of differences in population such as the number of dogs that had ileal and colonic evaluation, number of dogs that received prednisolone before admission, dosages and length of prednisolone administration. PON-1 activity was significantly decreased in iPLE dogs when compared with healthy dogs. Since the decrease in PON-1 activity seems to couple with inflammation, our results might suggest that oxidation and inflammation might occur in some dogs with iPLE [65]. During inflammation, one of the most consistent alterations is the reduced serum concentration of HDLs [66]. In our study, a negative correlation between serum CRP concentration and percentage of HDLs has been found, as previously observed [67]. Indeed, HDLs are thought to have anti-inflammatory properties [68]. One additional property of HDLs is to protect LDLs from oxidation, and several HDL-related proteins, such as ApoA1, PON-1 and transferrin could affect this important function [69,70]. The anti-oxidative function of HDLs is strongly associated with disease severity, while the use of anti-inflammatory treatments significantly restores the antioxidant functions of HDLs towards normal [71,72]. In this study, positive correlations were found between PON-1 activity concentrations and HDLs; meanwhile, negative correlations were found between PON-1 activity concentrations and LDLs, and between CRP concentrations and HDLs. Taken together, these results might support the hypothesis that changes in HDLs depend on oxidative stress, which is likely associated with inflammation. Therefore, similarly to human medicine, canine HDLs might have anti-inflammatory and anti-oxidative properties, while LDLs might play a pro-inflammatory role [67]. Furthermore, positive correlations between age and Chol, and body weight and Chol were found in iPLE dogs, suggesting that biochemical characteristics of lipid metabolism disorders may be affected by aging and weight [73]. Unlike the results that have been previously reported, no correlations were found between the lipid profile and BCS [74]. However, the subjective nature of the BCS method, as well as the assessors’ experience, might have influenced the results [75]. Furthermore, although BCS is commonly used in clinical practice, it might be inferior to other scores and techniques in accurately predicting the body fat composition [76,77].

Significant associations between the lipid profile and Alb were not found. However, when considering the hypoalbuminemia groups, Chol and HDLs were significantly lower in group C + D compared to group B, while VLDLs were significantly higher in group C + D compared to group B. These results are likely explained by the lipoprotein lipid content and function, along with inflammation, lymphangiectasia, fat malabsorption and malnutrition leading to hypocholesterolemia in dogs with iPLE. Indeed, HDLs are a major carrier of total circulating lipids, especially free and esterified cholesterol in dogs, while TGs account for a significant proportion of the lipids in VLDLs and LDLs, but not in HDLs [78]. 

No significant associations between the lipid profile and the mean cumulative lesion score were found. This result is not surprising since the mean cumulative lesion score might have been influenced by the lack of ileal and colonic mucosa evaluation in some iPLE dogs. However, since iPLE is a heterogeneous disorder and affected dogs might show different magnitudes of intestinal inflammation and lymphangiectasia, we compared the lipid profiles between dogs of the group 1 (inflammation with only mild or no lacteal dilation) and group 2 (inflammation with moderate or severe lacteal dilation). By this comparison, TGs were significantly higher in the dogs of the group 2. It can be hypothesized that these dogs with both inflammation and moderate or severe lacteal dilation, similarly to some patients with IBD, are characterized by a severe mucosal immune system dysregulation. This dysregulation leads to an increase in inflammatory cytokines that, in turn, may result in a decrease in lipoprotein lipase enzyme activity, leading to a characteristic lipoprotein profile with increased serum triglycerides and decreased HDLs [57].

In human patients with IBD, the administration of prednisone increases total Chol, HDLs and LDLs [20]. The increase in HDLs and LDLs can be explained by an increase in VLDL synthesis and lipoprotein lipase activity [79]. Moreover, glucocorticoids attenuate CRP induction and counteract oxidative stress by up regulating PON-1 gene expression [80,81]. In contrast, the concentration of CRP in dogs was not affected by the administration of oral corticosteroids for up to 3 weeks [82]. To date, glucocorticoids in addition to a dietary change seem to be appropriate for some dogs with CE and iPLE [11,57]. An exclusion criterion of this study was the use of immunosuppressive drugs within one month before admission. However, due to the widespread use of prednisolone, and the consequent difficulty in finding dogs that had never received it, dogs that received short-term, low-doses of prednisolone in the month before admission were included. Although some results here could have been explained by previous prednisolone administration, no significant differences in CRP and PON-1 activity concentrations, and lipid profiles were found between dogs that received prednisolone and dogs that did not. Although not expected, these results might have been influenced by the low number of dogs receiving prednisolone prior to admission, the low doses and the short length of administration. 

For dogs with iPLE and evidence of lymphangiectasia, low-fat diets alone or combined with prednisolone are proven to be useful [1,2,3,14,15,83,84]. However, to the best of the authors’ knowledge, no information exists on the influence of diets on lipoprotein profiles in dogs with iPLE. At the time of admission, the dogs of this study were fed different commercial and home-cooked diets, some of which were low in fat content, that had previously been selected on a case-by-case basis. Some canine limited-ingredient diets are fish- or vegetable-based diets, while highly digestible gastrointestinal diets are usually meat-based diets. Fish- and vegetable-based diets have been demonstrated to lower LDLs in dogs and humans, respectively [85,86]. No significant differences in the lipid profiles among our iPLE dogs assigned to different diet groups were found. However, these results might have been influenced by the type of grouping. Indeed, the diet subgroups were by diet type and not by the content of fat. Diet types have wide-ranging amounts of fat, and there is also wide variation in fat content among limited-ingredient diets and highly digestible gastrointestinal commercial diets.

This study included several limitations. First is the lack of information about the gold-standard method for lipoprotein evaluation. Without this information, the clinical utility of some data presented here cannot be interpreted reliably. Second, the results of the lipoprotein agarose gel electrophoresis are expressed as a percentage of the total area. Therefore, the increase in a lipoprotein class determines the reduction of another class, thus limiting the possibility of demonstrating an absolute increase in a lipoprotein class. Third, the diet subgroups were by diet type and not by content of fat, as already described above. This is mainly because the exact information about the brand of diets prescribed before admission was missing or not precise. Fourth, not all iPLE dogs had their ileal and colonic mucosa evaluated. This influenced the mean cumulative lesion score and potentially also the associations with the lipid profile. Fifth, the age of the control group was not paired with that of the iPLE dogs. It cannot be excluded that the unmatched age could have influenced the results. All animals of our control group were considered healthy based on the inclusion criteria reported above. At the time of inclusion, some healthy animals showed some values outside of the reference range. However, the magnitude of the increase was considered mild and clinically irrelevant. Moreover, values outside the reference range can be caused not only by pathologic conditions, but also by individual differences or physiologic conditions. For example, healthy dogs maintained in a normal environment had higher CRP concentrations (8.4 ± 4.6 mg/L) than dogs kept in a research “clean” environment (0.5 ± 0.2 mg/L) [87]. Exercise can also cause an increase in CRP concentration [88,89]. Finally, none of the control dogs developed any pathological conditions in the months following sampling. Although the magnitude of the increase was considered mild and clinically irrelevant, and it could be explained by individual differences or physiological conditions, subclinical diseases of healthy control dogs cannot be excluded. Sixth, one-quarter of the iPLE dogs received prednisolone, that is known to alter lipid metabolism. Although these dogs received low-dose, short-term administration of prednisolone, and their lipid profiles were not significantly different from those of dogs that had not received prednisolone, our results should be interpreted with caution. Finally, the storage stability of serum samples at −80 °C was not assessed. However, it was recently observed that canine serum lipoproteins are stable for several months when stored at −80 °C, similar to the storage of our samples [90]. 

## 5. Conclusions

Overall, lipid profile, CRP and PON-1 activity are altered in dogs with iPLE. High-density and low-density lipoproteins correlated with CRP and PON-1. The lipid profile did not vary as a function of disease severity; however, triglycerides were significantly higher in dogs with both intestinal inflammation and lymphangiectasia. Finally, in this population, low-dose, short-term administration of prednisolone in the previous month did not significantly influence the lipid profile. Additional investigation into the lipid metabolism of dogs with iPLE is warranted.

## Figures and Tables

**Table 1:** Comparative evaluation of the lipid profile, albumin, total proteins, C-reactive protein and paraoxonase-1 activity concentrations between healthy control dogs and dogs with inflammatory protein-losing enteropathy.

Parameter	Control Dogs	iPLE Dogs	Reference Interval	*p*-Value
Alb (g/dL)	3.6 (2.9–4.1)	1.7 (0.8–2.7)	2.8–4	<0.0001
Chol (mg/dL)	209.5 (118–289)	116 (59–327)	135–270	<0.0001
Chylomicrons (%)	2.1 (0.3–8.4)	5.6 (0.5–22.8)	-	<0.0001
CRP (mg/L)	0.9 (0–14.6)	5.4 (0–48.4)	≤5	=0.009
HDLs (%)	65.5 (44.4–79.5)	52.3 (18.7–81.8)	-	<0.0001
LDLs (%)	15.8 (6.5–39.1)	33.8 (13.7–64.3)	-	<0.0001
PON-1 activity (U/mL)	199 (129–303)	77 (13.3–223)	≥116	<0.0001
VLDLs (%)	15.6 (8.1–30.4)	6.6 (1.2–36.1)	-	<0.0001
TGs (mg/dL)	55 (11–122)	83 (30–172)	<104	<0.0001
TP (g/dL)	6.4 (4.8–8.9)	4.1 (2.0–7.4)	5.4–7.5	<0.0001

Data are reported as median (minimum–maximum). Alb = albumin; Chol = total cholesterol; CRP = C-reactive protein; HDLs = high-density lipoproteins; iPLE = inflammatory protein-losing enteropathy; LDLs = low-density lipoproteins; PON-1 = paraoxonase-1; VLDLs = very-low-density lipoproteins; TGs = triglycerides; TP = total protein.

**Table 2:** Significant correlations between the lipid profile and selected study variables among the 51 dogs with protein-losing enteropathy.

Pair of Variables Tested for Correlation	Correlation Coefficient (*r*)	Significance Level (*p*)
HDLs and PON-1	0.60	<0.0001
HDLs and CRP	−0.28	=0.048
LDLs and body weight	−0.36	=0.009
LDLs and PON-1	−0.68	<0.0001
Chol and age	0.30	=0.034
Chol and body weight	0.30	=0.039
Chol and PON-1	0.83	<0.0001
CRP and VLDLs	0.46	=0.001

Chol = total cholesterol; CRP = C-reactive protein; HDLs = high-density lipoproteins; LDLs = low-density lipoproteins; PON-1 = paraoxonase-1. *p* < 0.05 indicates a significant correlation. The strength of the correlations is categorized as follows: 0.00–0.39 weak correlation; 0.40–0.59 moderate correlation; 0.60–0.79 strong correlation; and 0.80–1.00 very strong correlation.

**Table 3:** Comparative evaluation of selected study variables and lipid profile between 13 dogs with inflammatory protein-losing enteropathy that received prednisolone before admission and 38 dogs with inflammatory protein-losing enteropathy that did not.

Parameter	iPLE Dogs P-Y	iPLE Dogs P-N	Reference Interval	*p*-Value
Age (months)	83 (24–119)	84.5 (19–171)	-	0.1147
Body weight (Kg)	13.4 (2.4–37.0)	16.7 (3.4–25.28)	-	0.6120
BCS	4 (2–6)	4 (1–6)	1–9	0.8039
CCECAI	9 (3–14)	8.5 (3–17)	0-≥12	0.6376
Alb (g/dL)	1.9 (1.1–2.7)	1.6 (0.8–2.5)	2.8–4	0.1610
Chol (mg/dL)	121 (66–327)	112.5 (59–266)	135–270	0.6422
Chylomicrons (%)	6.6 (1.7–14.2)	4.7 (0.5–22.8)	-	0.2355
CRP (mg/L)	4.4 (0–24.6)	5.9 (0–48.4)	≤5	0.5150
HDLs (%)	56.3 (26.9–81.8)	51.0 (18.7–71.2)	-	0.3817
LDLs (%)	33.8 (13.7–51.0)	34.8 (14.6–64.3)	-	0.2897
PON-1 activity (U/mL)	94.6 (33.6–220)	59.25 (13.3–223)	≥116	0.2030
VLDLs (%)	3.9 (1.9–36.1)	7.0 (1.2–24.6)	-	0.1167
TGs (mg/dL)	74 (30–172)	83 (47–157)	<104	0.8319

Data are reported as median (minimum–maximum). Alb = albumin; BCS = body condition score; Chol = total cholesterol; CCECAI = canine chronic enteropathy clinical activity index; CRP = C-reactive protein; P-Y = prednisolone yes; HDLs = high-density lipoproteins; iPLE = inflammatory protein-losing enteropathy; LDLs = low-density lipoproteins; PON-1 = paraoxonase-1; P-N = no prednisolone; VLDLs = very-low-density lipoproteins; TGs = triglycerides.

## Data Availability

All data analyzed during this study are included in this published article.

## Supplementary Materials

The following supporting information can be downloaded at: https://www.mdpi.com/article/10.3390/ani14213119/s1, Table S1: Correlations of the lipid profile with age, body weight, body condition score, CCECAI score, albumin, C-reactive protein, and paraoxonase-1 activity concentrations among dogs with inflammatory protein-losing enteropathy; Figure S1: Lipoprotein gel analysis of both healthy (A) and inflammatory protein-losing enteropathy dogs (B).
