# Supplementary material for: Comparative Evaluation of Lipid Profile, C-Reactive Protein and Paraoxonase-1 Activity in Dogs with Inflammatory Protein-Losing Enteropathy and Healthy Dogs
Source: Animals (Basel). 2024 Oct 29;14(21):3119. doi: 10.3390/ani14213119 (PMC11545359; doi:10.3390/ani14213119)
Supplement: Supplementary file 1 [file animals-14-03119-s001.zip › Table S1.pdf]

Table S1: correlation of the lipid profile with age, body weight, body condition score, CCECAI score, albumin, C-reactive protein, and Paraoxonase-1 activity concentrations among dogs with inflammatory protein-losing enteropathy

		Chol	TG	HDL	VLDL	LDL	Chylomicrons
**Age**	*r*	0.30	0.04	0.17	0.15	-0.19	-0.14
	p	0.034*	0.773	0.240	0.298	0.193	0.312
**Weight**	*r*	0.29	-0.12	0.26	0.27	-0.36	-0.07
	p	0.039*	0.398	0.063	0.059	0.009*	0.616
**BCS**	*r*	-0.19	0.02	-0.21	-0.01	0.22	0.10
	p	0.251	0.895	0.197	0.929	0.174	0.562
**CCECAI**	*r*	-0.20	0.04	-0.23	0.19	0.21	-0.07
	p	0.163	0.771	0.101	0.180	0.149	0.601
**Albumins**	*r*	0.04	-0.18	0.12	-0.01	-0.19	0.09
	p	0.780	0.205	0.386	0.923	0.186	0.513
**CRP**	*r*	0.19	0.19	-0.28	0.46	0.14	-0.31
	p	0.175	0.181	0.048*	0.001*	0.316	0.026*
**PON-1**	*r*	0.84	-0.15	0.60	0.03	-0.68	0.02
	p	<0.0001*	0.279	<0.0001*	0.834	<0.0001*	0.880

BCS= body condition score; CRP= C-reactive protein; PON-1=paraoxonase-1 activity
