# Supplementary figures and images for: Comparative Evaluation of Lipid Profile, C-Reactive Protein and Paraoxonase-1 Activity in Dogs with Inflammatory Protein-Losing Enteropathy and Healthy Dogs
Source: Animals (Basel). 2024 Oct 29;14(21):3119. doi: 10.3390/ani14213119 (PMC11545359; doi:10.3390/ani14213119)

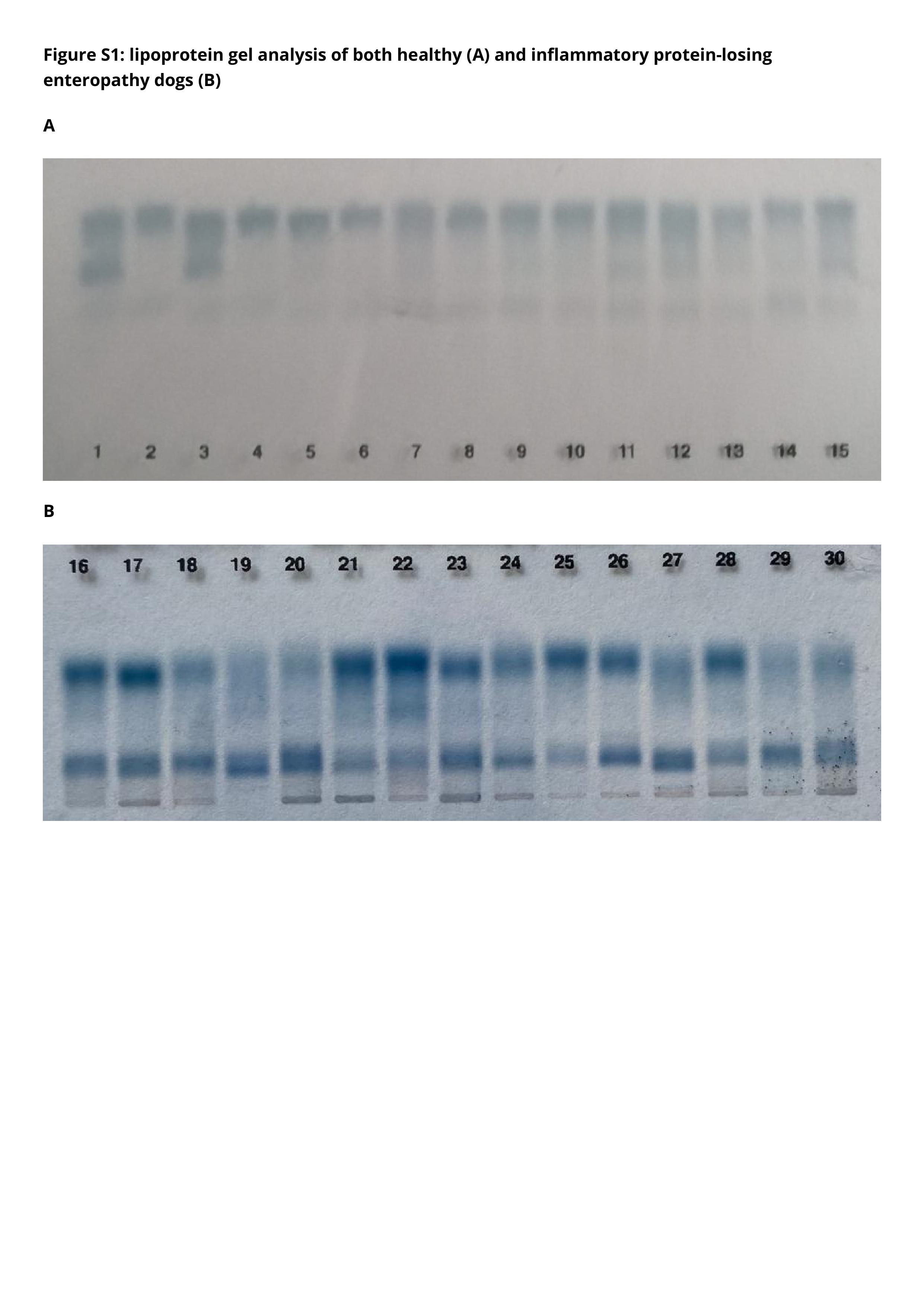

Supplement: Supplementary file 1 [file animals-14-03119-s001.zip › Figure S1.jpg]
